# Tunneled and Non-tunneled Epidural Catheters for Acute Post-surgical Pain

**DOI:** 10.7759/cureus.103832

**Published:** 2026-02-18

**Authors:** Kia Lor, Anthony Stephenson, Kalli Fautsch

**Affiliations:** 1 Anesthesiology, Mayo Clinic, Rochester, USA

**Keywords:** acute non-surgical pain, epidural infection risk, neuraxial analgesia, opioid tolerance, tunneled epidural catheter

## Abstract

Multimodal analgesia, particularly neuraxial techniques, is vital for managing perioperative pain in opioid-tolerant patients. This report describes the case of a 34-year-old woman with a complex surgical history and opioid tolerance with an indwelling tunneled thoracic epidural catheter who subsequently underwent placement of a second non-tunneled lumbar catheter for surgical pain control. The dual epidural catheters, in addition to an optimized multimodal regimen, achieved significant postoperative pain control while minimizing systemic opioid escalation. This case highlights the value of neuraxial techniques in managing refractory perioperative pain in complex, opioid-tolerant patients and underscores current knowledge about infectious risks associated with neuraxial catheters.

## Introduction

Neuraxial analgesia is a widely utilized technique for the management of surgical pain and is considered a standard of care for thoracic and abdominal surgeries [1,2]. It offers several benefits, including reduced postoperative pulmonary complications, decreased systemic opioid requirements, decreased incidence of deep venous thrombus, and fewer overall systemic analgesic-related adverse effects [3]. However, neuraxial analgesia is not without risks. Potential complications include neuraxial bleeding, infection, unintentional dural puncture, neurologic injury, and failure of placement [4].

Traditionally, epidural catheters are placed in the thoracolumbar spine for acute surgical pain control, with the specific level of the placement depending on the anticipated location of postoperative pain. These catheters are secured with external devices such as clips or adhesive dressings to prevent accidental withdrawal. Limitations to epidural catheters are their short lifespan due to time-related risk of infection and limited dermatomal coverage of pain due to reliance on the spread of medication. To combat these limitations, tunneled epidural catheters or multiple epidural catheters have been utilized for the treatment of cancer pain and in extensive thoracoabdominal surgeries, respectively [5]. However, these variations of traditional techniques are uncommon. In this case report, we present a medically complex opioid-tolerant patient with chronic abdominal pain who underwent a lumbar epidural catheter placement in addition to a preexisting indwelling tunneled thoracic epidural catheter for acute surgical pain control.

## Case presentation

**Figure 1:**
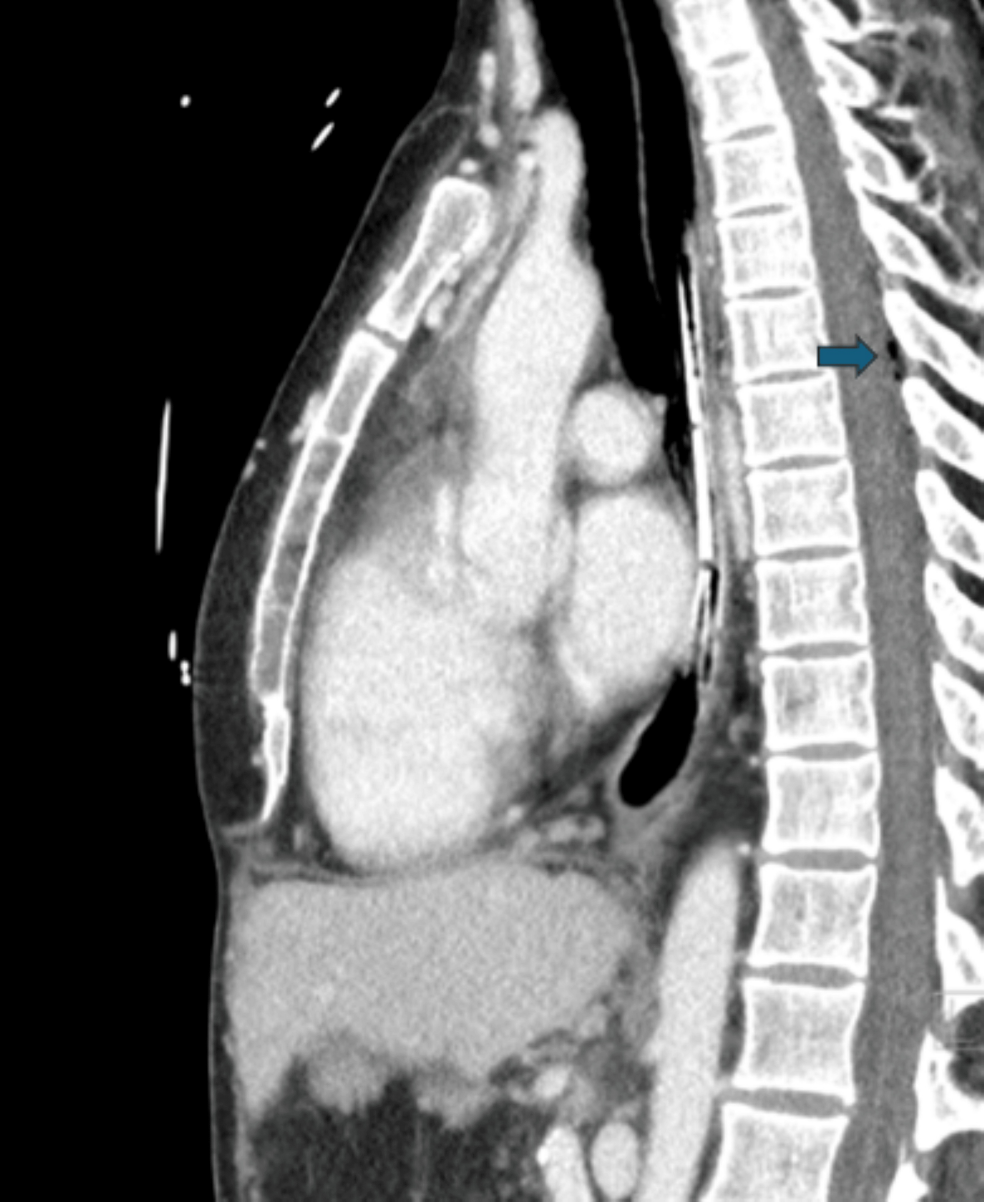
Computed tomography of the thoracic spine without contrast demonstrating epidural infusion at T7-T8

The patient is a 34-year-old woman with an extensive medical history notable for Ehlers-Danlos syndrome, esophageal dysmotility, diaphragmatic hernia status post (s/p) multiple open repairs with mesh placement, gastric volvulus s/p Roux-en-Y, malnutrition requiring parenteral nutrition, and chronic abdominal pain on chronic opioids. The outpatient pain regimen consists of methadone 20 mg three times a day and oxycodone immediate-release 20 mg every four hours as needed (prn) with a reported baseline chronic pain score of 4/10 on the numerical rating scale (NRS). The patient was transferred from an outside hospital for surgical planning for recurrent bowel obstruction secondary to mesh dysfunction. The Inpatient Pain Service was consulted for the management of acute obstruction-related pain and for assistance with an indwelling tunneled thoracic epidural catheter placed five weeks prior at the previous institution. The tunneled epidural was placed at T7-T8 and was infusing bupivacaine 2 mg/mL, fentanyl 5 mcg/mL, and epinephrine 2 mcg/mL at 5 mL/hr with 3 mL patient-controlled epidural analgesia capabilities (Figure 1). Upon admission, the infusion was replaced with fentanyl 5 mcg/mL and bupivacaine 0.075% at a rate of 5 mL/hr with boluses of 3 mL available every 15 minutes. The neuraxial analgesia was combined with several systemic adjuncts including intravenous acetaminophen 1 g every six hours, transdermal clonidine 0.3 mg per day, intravenous ketamine infusion at 0.2 mg/kg/hr, intravenous diazepam 2.5 mg every 12 hours prn, intravenous hydromorphone boluses of 10 mg every four hours prn, and intravenous methadone 45 mg twice a day and 50 mg at nighttime. Her total opioid requirement was approximately 5,500 morphine milligram equivalents (MME), with a reported NRS pain score of 2/10.

On hospital day 7, the patient underwent exploratory laparotomy, esophagogastroduodenoscopy, and drain placement. Intraoperatively, the tunneled thoracic epidural was increased to 8 mL/hr, and intravenous ketamine infusion was increased to 0.3 mg/kg/hr, with a total of 85 mg of intravenous ketamine and 62 mg of intravenous hydromorphone administered. Postoperatively, the patient reported uncontrollable pain, 10/10 on the NRS, and was admitted to an intensive care unit for pain control. A dexmedetomidine infusion was initiated and quickly titrated to 1.5 mcg/kg/hr, resulting in improved pain control and a reported score of 7/10.

On hospital day 9, the surgical team discussed plans for a defunctioning esophagectomy and jejunostomy. Due to inadequate pain control with intravenous analgesics and the tunneled epidural catheter, a second epidural catheter was discussed. The patient underwent placement of a second epidural at the L3-L4 interspace that was infused with bupivacaine 0.075% at 5 mL/hour in addition to the thoracic epidural infused with both fentanyl 5 mcg/mL and bupivacaine 0.075% at 8 mL/hr. Intraoperatively, intravenous ketamine infusion was increased to 0.4 mg/kg/hr to assist with pain control. Postoperatively, the patient was kept intubated and sedated on a propofol infusion at 50-80 mcg/kg/hr and a hydromorphone infusion at 5 mg/hr. The next day, the propofol infusion was weaned, and a bolus of 3 mL of bupivacaine 0.075% was administered through the lumbar catheter prior to extubation. Immediate assessment revealed a reported pain score of 3-4/10 with left lower extremity weakness thought to be related to the infusion of bupivacaine. The lumbar epidural infusion was discontinued the following day without inciting a pain crisis.

On hospital day 12, with reported pain scores of 5-6/10, weaning of infusions was initiated in anticipation of transfer to inpatient floor care. The tunneled thoracic epidural concentrations were increased to hydromorphone 10 mg/mL and bupivacaine 0.1%, and the rate increased to 12 mL/hr. Two days later, the patient developed low-grade fevers with a temperature of approximately 38°C and a leukocytosis with a white blood cell count of 16.2×10^9^/L. Physical exam showed erythema around the tunneled epidural skin insertion site despite lack of pain at the site. These findings prompted the removal of the thoracic epidural. The patient's pain remained at 5-6/10 post-removal, and she was continued on a multimodal pain regimen. 

**Figure 2:**
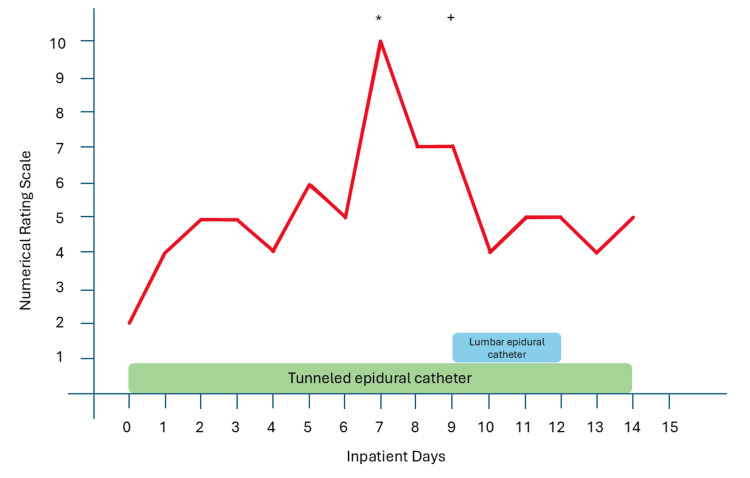
Course of inpatient treatment and pain scores *: exploratory laparotomy, esophagogastroduodenoscopy, and drain placement; +: defunctioning esophagectomy and jejunostomy

Figure 2 depicts the course of inpatient treatment and pain scores.

## Discussion

Effective pain control is an important component of postoperative recovery. It facilitates early ambulation, shortens hospital stay, and reduces respiratory complications [6]. Multimodal regimens with both pharmacologic and nonpharmacologic modalities are critically important in reducing opioid requirements and associated side effects. This is particularly important in treating patients with chronic opioid use with tolerance, hyperalgesia, and allodynia at baseline [1]. This report presents a case where neuraxial analgesia through dual epidural catheters, one tunneled and one non-tunneled, assisted in acute surgical pain in a medically complex and opioid-tolerant patient following an esophagectomy.

Regional analgesia is vital in the management of perioperative pain with its specific target of dermatomes [2]. Analgesia is achieved via peripheral uptake or cerebral spinal fluid absorption of medications, allowing for effective pain control while reducing opioid requirements [2]. When a procedure involves both the thoracic and abdominal regions, such as esophagectomies, the spread of anesthetics from a single epidural catheter may be inadequate for coverage. Neuraxial analgesia via dual epidural catheters addresses this limitation by providing more extensive dermatomal coverage. Brown et al. found that patients with dual epidural catheters following esophagectomies had significantly reduced pain with movement on postoperative days 0-3, halved the combined rate of major postoperative complications, and increased hospital-free days compared to single-catheter patients [5]. Similar outcomes have been observed in open colorectal surgeries [7].

While uncommon in the United States, tunneled epidural catheters are frequently used internationally for palliative pain control. A retrospective analysis of 25 pediatric patients in whom tunneled epidural catheters were placed for palliative pain showed that tunneled catheters were safe and effective while reducing systemic opioid requirement when conventional analgesics failed or were found to be impractical [8]. Similarly, another study showed that tunneled catheters reduced oral morphine equivalents by 122.73 mg in hospice patients [9]. Despite both studies underscoring the benefits of tunneled catheters in the management of intractable chronic pain, their application in postoperative pain remains underexplored.

Neuraxial techniques are minimally invasive procedures that, while effective, are associated with the risk of infection. Balancing the potential therapeutic benefits with the risk of infection is an important component of the informed consent process for neuraxial analgesia. Common infection sources include skin flora contamination at the catheter exit site, insertional contamination, or hematogenous spread from wounds or the bloodstream [5]. Hayek et al. examined 260 tunneled epidural catheters placed in 218 patients over 10,985 catheter-days and found 24 epidural space infections and 34 suspected superficial infections [10]. Another study on epidural catheters with a duration of placement greater than seven days for cancer pain suggests that one in 35 individuals with an epidural catheter for 74 days was expected to have a deep epidural infection, with one in 500 likely to die of infection-related causes [11]. Similarly, a literature review by Selvamani et al. looking at infectious complications following neuraxial anesthesia found the rate of any infectious complication to be nine out of 100,000, reinforcing that the risk of infection is low [12].

The low risk of infection associated with neuraxial analgesia may be attributed to strict aseptic precautions and current technique standards as endorsed by the American Society of Anesthesiologists and the American Society of Regional Anesthesia and Pain Medicine [13]. To our knowledge, both the tunneled and non-tunneled catheters in this case were placed using strict aseptic techniques. Although the patient likely developed a localized skin infection at the tunneled epidural insertion site necessitating immediate removal, this was most likely related to the prolonged infusion of more than seven weeks. Additionally, existing literature suggests that infection risk may remain low even when strict aseptic adherence is not feasible. Field conditions, such as those in battlefield and disaster zones, present unique challenges that often necessitate deviations from standard protocols. An analysis of peripheral nerve catheters and epidural catheters in wounded soldiers with combat injuries sustained in Iraq and Afghanistan reported a 1.9% infection rate and an 11.9% catheter-related complication [14]. Notably, no infection was reported among 50 catheters with a duration of placement from two to 17 days at the time of publication, underscoring the potential safety use of extended catheter duration even under suboptimal conditions.

## Conclusions

Neuraxial analgesia is an essential component of perioperative pain management, particularly in thoracoabdominal surgeries and in complex, opioid-tolerant patients without contraindications. In patients with significant opioid tolerance, it plays a critical role in establishing effective multimodal analgesia. By providing effective pain control, neuraxial techniques lead to a variety of benefits, including reduced pulmonary complications, reduced opioid requirements and escalation, and fewer systemic analgesic-related adverse effects. While the risk of infection must be weighed, the benefits underscore its value. Current literature suggests that the overall risk of infection associated with tunneled epidural catheters may be low, with skin infection being the most common. Additional research on its safety use, particularly time-dependent infection risks associated with dual and tunneled catheters, may further clarify best practices and expand its utility across multiple clinical settings.
